# Liraglutide Attenuates Diabetic Cardiomyopathy via the ILK/PI3K/AKT/PTEN Signaling Pathway in Rats with Streptozotocin-Induced Type 2 Diabetes Mellitus

**DOI:** 10.3390/ph17030374

**Published:** 2024-03-15

**Authors:** Shatha M. Alobaid, Rahaf M. Alshahrani, Asma S. Alonazi, Nawal M. Alrasheed, Maha A. Alamin, Tahani K. Alshammari, Anfal F. Bin Dayel, Doaa M. Elnagar, Rana R. Alotaibi, Lama A. Almuthnabi, Dalia H. Almasud, Shahad E. Al-Ammar, Shahad O. Almadhi, Reema A. Almalke, Nouf T. Aldamri, Hanan K. Alghibiwi, Dalal A. Alkhelb, Nouf M. Alrasheed

**Affiliations:** 1 PharmD Program, College of Pharmacy, King Saud University, Riyadh 11451, Saudi Arabia; shathamajed4@gmail.com (S.M.A.); rahaf11301@gmail.com (R.M.A.); 441200367@student.ksu.edu.sa (R.R.A.); 441200476@student.ksu.edu.sa (L.A.A.); 442200723@student.ksu.edu.sa (D.H.A.); 441200489@student.ksu.edu.sa (S.E.A.-A.); 441200322@student.ksu.edu.sa (S.O.A.); 439201033@student.ksu.edu.sa (R.A.A.); 2 Department of Pharmacology and Toxicology, College of Pharmacy, King Saud University, Riyadh 11451, Saudi Arabia; aaloneazi@ksu.edu.sa (A.S.A.); nalrasheed@ksu.edu.sa (N.M.A.); mahaali@ksu.edu.sa (M.A.A.); talshammary@ksu.edu.sa (T.K.A.); abindayel@ksu.edu.sa (A.F.B.D.); naldamri@ksu.edu.sa (N.T.A.); halghibiwi@ksu.edu.sa (H.K.A.); dalkhelb@ksu.edu.sa (D.A.A.); 3 Department of Zoology, College of Science, King Saud University, Riyadh 11451, Saudi Arabia; elnagard1@yahoo.com; 4 Department of Zoology, Faculty of Woman, Ain Shams University, Cairo 11566, Egypt

**Keywords:** glucagon-like peptide-1 analog, diabetic cardiomyopathy, streptozotocin-induced type 2 diabetes, integrin-linked kinase, phosphatidylinositol 3-kinase, protein kinase B

## Abstract

One of the possible candidates for the treatment of diabetic cardiomyopathy is liraglutide, a glucagon-like peptide-1 receptor (GLP1R) agonist. In this study, the impacts of liraglutide on the integrin-linked kinase (ILK)-related PI3K/AKT axis in rats with type 2 diabetes induced via streptozotocin were examined. Twenty-four Wistar albino rats were distributed in four different groups, and a high-fat diet and streptozotocin were used to induce type 2 in two groups. Rats in the untreated control groups were administered 0.9% NaCl solution over a 6-week period, and those in the treatment groups were administered 0.9% NaCl for 3 weeks, followed by subcutaneous injection of liraglutide (150 μg/kg) for an additional 3 weeks. In the liraglutide-treated diabetic group, the heart-to-body weight ratio was significantly reduced, levels of cardiac biomarkers, troponin I and creatine-kinase-MB, were improved; activities of antioxidant enzymes, glutathione peroxidase and superoxide dismutase, were increased; and levels of malondialdehyde were decreased. Western blotting and immunohistochemical studies revealed increased levels of ILK, P-PI3K, P-AKT, and BCL2, as well as those of caspase 3, BAX, and P-PTEN, indicating mitigation of cardiomyocyte apoptosis. Our results show that liraglutide, by targeting GLP1Rs, enhances the expression of proteins in the ILK/PI3K/AKT/PTEN pathway and thereby exerts its cardioprotective effects in rats with DCM.

## 1. Introduction

One of the features of diabetes mellitus is that the levels of blood glucose are raised as a result of a decline in the secretion or activity of insulin, which is a significant global health concern [1]. Recent World Health Organization reports (2000–2021) place diabetes among the top 10 causes of death and disability worldwide, with current estimates of 451 million people affected and 693 million people predicted to be affected by 2045 [2,3]. Type 2 diabetes mellitus (T2DM), which has assumed the proportions of a pandemic, is associated with a substantially increased risk of cardiovascular diseases. Individuals with T2DM have twice the risk of cardiovascular complications compared to their nondiabetic counterparts [4]. The initial manifestation of diabetic cardiomyopathy (DCM) is impaired diastolic relaxation, which ultimately leads to clinical heart failure, even when coronary artery disease, hypertension, or dyslipidemia are not present.

Insulin resistance, hyperinsulinemia, and hyperglycemia are closely associated with the development of DCM and have been identified as independent risk factors [5]. Impaired insulin signaling in the heart results in changes in its functioning and structure, contributing to DCM [5]. Inappropriate activation of the renin–angiotensin–aldosterone system is postulated to be a key mechanism in cardiac insulin resistance, which promotes insulin resistance via an increase in the phosphorylation of serine residues in insulin receptor substrate 1 (IRS1), leading to the impairment of PI3K engagement and AKT stimulation [6]. When the physiological conditions are considered normal, the PI3K/AKT signaling pathway in cardiomyocytes enables glucose transporter type 4 (GLUT4) to be recruited to the plasma membrane, mediating the uptake of glucose into the heart [6,7]. GLUT4 plays a critical factor in the process by which insulin resistance develops, as well as how glucose homeostasis is regulated in general [8]. Furthermore, the PI3K/AKT pathway contributes significantly to cardiomyocyte survival and functions downstream of insulin signaling [9]. Extensive evidence supports the role of insulin in promoting cell survival through PI3K/AKT activation [10]. Additionally, together with selenium, insulin was reported to regulate glycemia and restore the distribution of GLUT4 in the cardiac muscles of diabetic rats via an IRS1/PI3K-dependent pathway [11]. Consistent with PI3K-dependent activation, integrin-linked kinase (ILK) signaling is negatively regulated by phosphatase and the tensin homolog (PTEN). Cardiac-specific knockout of PTEN protected the heart from cardiac dysfunction, probably through the upregulation of ILK and AKT [11,12,13].

The serine/threonine protein kinase, ILK, is bound to β-integrins’ cytoplasmic tail, thus establishing a connection between cell–matrix interactions and signals responsible for regulating different processes in the cell [14]. Its expression is the highest in the heart, and alterations in its activity can lead to cardiomyopathy or sudden death [15]. As a result of the upregulation of ILK, compensatory hypertrophy is induced in mouse models, whereas loss-of-function mutations or absence of expression in humans can lead to dilated cardiomyopathy syndrome [15]. After myocardial infarction, activation of ILK promotes cardiac repair, suggesting a potential target for therapeutic interventions [16]. Considering the central role of ILK in cardiac physiology, further studies may unravel its importance in the maintenance of heart health. ILK activity can be stimulated by growth factor stimulation and matrix attachment in a PI3K-dependent manner [17], as well as by PKB/AKT phosphorylation [18]. Targeted ILK deletion in the murine heart has been linked to dilated cardiomyopathy and spontaneous heart failure [19].

Although current medications aim to improve cardiac complications of diabetes, the residual risk persists, leading to the progression of heart failure in some patients. Three new targets for antidiabetic agents, glucagon-like peptide 1 receptor (GLP1R), dipeptidylpeptidase 4 (DPP4), and sodium-glucose cotransporter 2 (SGLT2), have gained popularity because of their crucial role in DCM [20]. However, the mechanisms underlying the attenuation of DCM remain unclear. Among these targets, GLP1Rs are expected to become the preferred drug targets for diabetes and its associated cardiac complications in the future, surpassing current options owing to their specificity, lower toxicity, and ability to overcome drawbacks, such as weight gain and hypoglycemia [21].

GLP1 agonists, including liraglutide, exhibit significant cardiovascular benefits and have been approved for diabetes treatment [22]. Several animal studies have shown that liraglutide can attenuate systolic and diastolic dysfunction in rodents with type 2 diabetes [23,24]. Furthermore, liraglutide has been reported to ameliorate left ventricular dysfunction and reduce the heart rate in diabetic rats [25]. Liraglutide, a human GLP1 analog, has gained attention for its efficacy in the treatment of T2DM without causing major renal damage or increased risk of hypoglycemia [26]. The LEADER trial highlighted lower rates of cardiovascular death in patients with T2DM on liraglutide [26]. This trial also unraveled cardiovascular benefits, including a reduction in the risk of sudden cardiac death, nonfatal myocardial infarction, stroke, and death from any cause, as well as reduced hospitalization due to congestive heart failure [27]. It was demonstrated that subcutaneous GLP1 infusion resulted in improvements in survival rates, remodeling, and cardiac function in rats diagnosed as having chronic heart failure [28]. Liraglutide has also been explored as a potential cognitive enhancer in the hippocampus of Goto–Kakizaki rats with type 2 diabetes mellitus. In this study, the Morris Water Maze test was utilized to assess the impact of low and high doses of liraglutide on learning and memory in diabetic rats. The authors also investigated the molecular mechanism underlying liraglutide’s neuroprotective effect and reported that it increased the expression of the mammalian target of rapamycin (mTOR) in hippocampus tissue in diabetic rats via the AMP-activated protein kinase (AMPK) and PI3K/AKT pathways [29]. An in vitro study using H9c2 cells demonstrated that GLP-1R agonists protected cardiomyocytes against apoptosis (measured using TUNEL assay), and the protective effect attenuated by PI3K and/or extracellular signal-regulated kinase 1/2 (ERK1/2) inhibition was confirmed via an immunoblotting assay [30].

Although GLP1R and liraglutide are considered promising DCM treatments owing to their safety and effectiveness, the exact mechanism by which liraglutide improves cardiovascular outcomes is not fully understood. Despite the crucial roles played by the ILK and PI3K/AKT signaling pathways in cardiac survival, growth, function, and repair, attenuation of DCM by liraglutide via the ILK and PI3K/Akt/PTEN axis has not been confirmed. We hypothesized that liraglutide attenuates DCM by modulating the ILK/PI3K/AKT signaling pathway and conducted this study to test our hypothesis in rats with streptozotocin (STZ)-induced T2DM.

## 2. Results

### 2.1. Liraglutide Treatment’s Effects on Body Weight, Blood Glucose, and Heart Weight-to-Body Weight Ratio in Rats with Diabetic

Subsequent to treating the rats over a period of six weeks, measurements were taken to determine the levels of blood glucose, the ratio, as well as body weight (Table 1). Compared with the control group rats, those in the diabetic group had levels of blood glucose that were significantly higher (*p* < 0.001). As a result of being treated with liraglutide, rats with diabetes exhibited a marked decrease in blood glucose levels in comparison with rats in the control group with no diabetes. Notably, the body weight of diabetic rats after liraglutide treatment was not significantly lower than that of untreated diabetic rats. The heart weight-to-body weight ratio, which often indicates a hypertrophied heart, was significantly increased in the control group of diabetic rats compared with that in the normal control group (*p* < 0.001). The heart weight-to-body weight ratio (*p* < 0.001) in rats with diabetes was remarkably reduced after treatment with liraglutide compared with that in untreated diabetic control rats. Conversely, glucose levels, body weight, and heart weight-to-body weight ratio in the liraglutide-treated healthy (nondiabetic) group were not significantly reduced (Table S1A–C).

### 2.2. Effect of Liraglutide on Cardiac Marker Enzymes in the Serum

**Figure 1:**
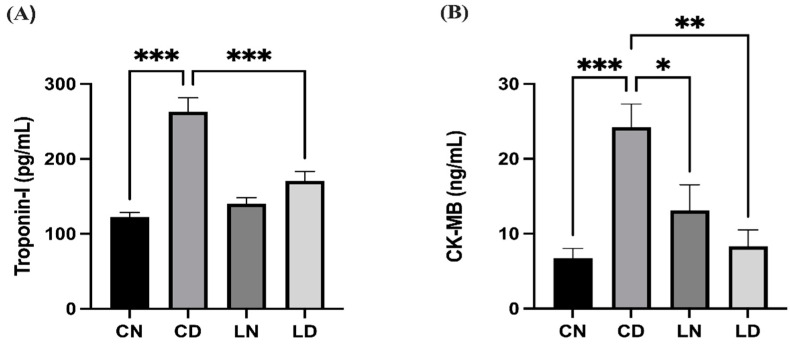
Liraglutide treatment effect on serum cardiac biomarkers in rats with diabetes. (**A**) Troponin I levels. (**B**) Creatine kinase-MB (CK-MB) levels. Data are presented as means ± standard error of the mean (SEM) (*n* = 6 samples per group). One-way analysis of variance (ANOVA) was performed for comparison of data, followed by the Tukey–Kramer post hoc test. CN, normal control group; CD, diabetic control group; LN, nondiabetic group treated with liraglutide; LD, diabetic group treated with liraglutide. Significant differences are denoted as *** *p* < 0.001 in comparison to the CN group; * *p* < 0.05, ** *p* < 0.01, *** *p* < 0.001 in comparison to the CD group.

Diabetic rats in the control group had significantly elevated levels of serum troponin I and creatine kinase-MB (CK-MB) in comparison with control group rats without diabetes (*p* < 0.001), suggesting that the heart musculature had potentially been damaged. Conversely, the serum levels of troponin I and CK-MB in rats in the group that received liraglutide treatment were notably lower than in control group rats with diabetes (*p* < 0.001 and *p* < 0.01, respectively) (Figure 1) (Table S1D,E).

### 2.3. Effect of Liraglutide on the Heart of Diabetic Rats Evaluated Using H&E-Stained Heart Sections

**Figure 2:**
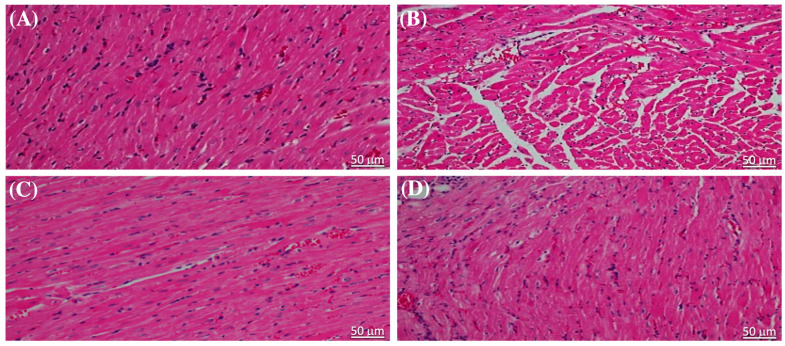
Liraglutide alleviated the effect of streptozotocin on the cardiac structure of the heart tissue of rats. The hematoxylin and eosin-stained heart tissue sections from (**A**) rats in the normal control group and (**B**) in normal rats that were administered liraglutide showing typical histological features of myocardial cell cytoplasm and nuclei. (**C**) Myocardial cells in sections from diabetic rats showing cytoplasm degeneration and pyknotic nuclei. (**D**) Sections from diabetic rats that were administered liraglutide showing lesser degeneration of cytoplasm and nuclei in the myocardial cells. Scale bar = 50 μm (original magnification, ×50).

The results of the evaluation of serum cardiac biomarkers, which are indicative of cardiac injury caused by STZ, were corroborated through histopathological examination of sections of heart tissue stained with hematoxylin and eosin (H&E). The cardiac muscle structure in normal, healthy rats was distinctly affected by liraglutide treatment (Figure 2A,B). Notably, liraglutide treatment in diabetic rats resulted in reduced myocardial degeneration compared with that in the diabetic control (Figure 2C,D).

### 2.4. Liraglutide Treatment Effect on Cardiac Oxidative Stress Biomarkers in Rats with Diabetes

**Figure 3:**
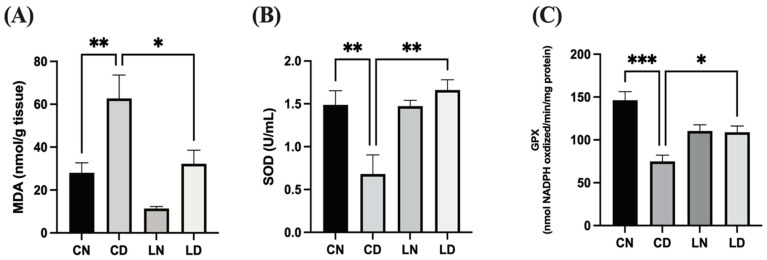
Liraglutide treatment effects on cardiac oxidative stress markers in rats with diabetes. (**A**) Malondialdehyde (MDA) levels. (**B**) Superoxide dismutase (SOD) activity. (**C**) Glutathione peroxidase (GPx) activity. Data are exhibited in the form of means ± SEM (*n* = 6 samples in each group). One-way ANOVA was used for inter-group comparisons, then the Tukey–Kramer post hoc test. CN, normal control group; CD diabetic control group; LN, nondiabetic rats receiving liraglutide treatment; LD, diabetic rats receiving liraglutide treatment. Differences reaching a level of significance are indicated as ** *p* < 0.01 and *** *p* < 0.001 compared with the normal control group (CN), and * *p* < 0.05; ** *p* < 0.01 compared with the control group containing diabetic rats (CD).

To assess the antioxidative effect of liraglutide in diabetic rats, we performed an enzyme-linked immunosorbent assay (ELISA) to examine malondialdehyde (MDA) levels and superoxide dismutase (SOD) and glutathione peroxidase (GPx) activities in the myocardial tissue. Diabetic rats in the control group exhibited notably elevated levels of MDA (*p* < 0.01) (Figure 3A), while SOD and GPx activities were detected to be markedly reduced in comparison with rats in the control group *(p* < 0.01 and * *p* < 0.001, respectively) (Figure 3B,C). The levels of MDA in rats with diabetes induced via STZ that subsequently received liraglutide treatment were lowered (*p* < 0.05), whereas SOD (*p* < 0.01) and GPx (*p* < 0.05) activities were elevated (Figure 3). These findings suggest that liraglutide treatment has the potential to alleviate diabetes-induced oxidative stress in the heart tissue (Table S1F–H).

### 2.5. Liraglutide Treatment’s Effects on Cardiomyocyte Apoptosis in Rats with Diabetes

**Figure 4:**
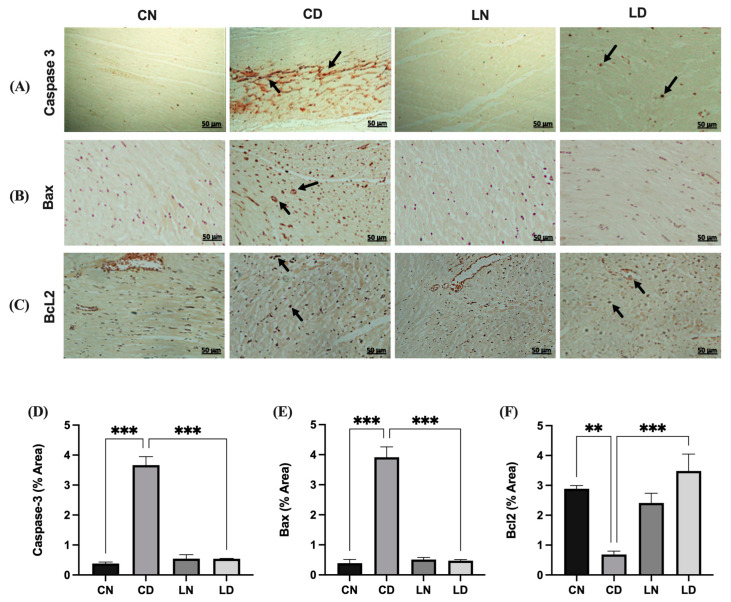
Liraglutide treatment’s effects on caspase 3, BAX, and Bcl2 expression in the heart tissue of rats with diabetes. Photomicrographs showing caspase 3 (**A**) and BAX (**B**) immunostaining of the heart sections: (CN) shows normal myocardium cytoplasm and nuclei, (CD) shows strong immunoreactivity in myocardium cell nuclei and moderate immunoreactivity in the cytoplasm (black arrow), (LN) exhibits lack of immunopositive cytoplasm and nuclei, and (LD) shows reduced and few immunopositive nuclei. Bcl2 (**C**): (CN) shows normal myocardium cytoplasm, (CD) shows strong immunoreactivity in myocardium cell nuclei and moderate immunoreactivity in the cytoplasm, (LN) exhibiting the absence of immunopositive cytoplasm, and (LD) shows a significant reduction in the expression and few immunopositive nuclei. Scale bar = 50 μm; magnification, ×20. Semi-quantification results for caspase 3 (**D**), BAX (**E**), and Bcl2 (**F**) immunoreactivity (using ImageJ software, version 1.8.0_172). Data are expressed as mean ± SEM (*n* = 3 different analyses per group). Statistical analyses were performed using one-way ANOVA; ** *p* < 0.01, *** *p* < 0.001 compared to CD. Abbreviations: CN: normal control rats; CD: diabetic control rats; LN: liraglutide-treated normal rats; LD: liraglutide-treated diabetic rats.

To evaluate the potential antiapoptotic effects of liraglutide in diabetic rats, we determined the expression of caspase 3, BAX, and BCL2 immunohistochemically (Figure 4A–C). In the diabetic rat group, the expression of BAX and cleaved caspase 3 was increased compared with that in the control group (Figure 4A,B). Liraglutide treatment mitigated the increase in the expression of BAX and cleaved caspase 3 in the heart tissue of diabetic rats compared with that in diabetic control rats (Figure 4A,B). Conversely, the expression of the antiapoptotic marker BCL2 increased in diabetic rats treated with liraglutide compared with that in rats in the diabetic control group (Figure 4C).

### 2.6. Effect of Liraglutide on Cardiac Cell Apoptosis in Diabetic Rats Evaluated Using the TUNEL Assay

**Figure 5:**
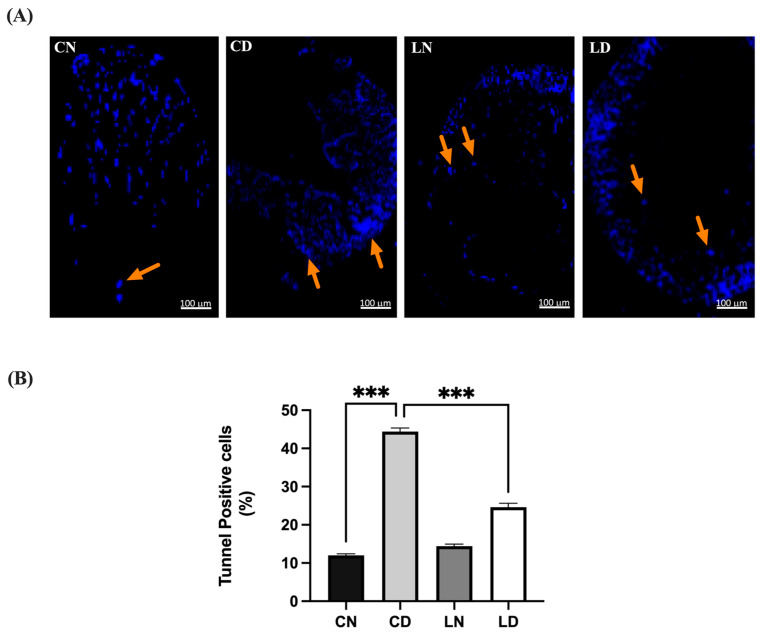
Liraglutide treatment’s effect on myocardial apoptosis in rats with type 2 diabetes induced via STZ/high-fat diet (HFD). (**A**) Photomicrographs showing TUNEL staining of the heart tissue sections from normal control rats (CN), diabetic control rats (CD), liraglutide-treated normal rats (LN), and liraglutide-treated diabetic rats (LD). TUNEL-positive cells are indicated by orange arrows and are displayed in light blue (original magnification, ×100). (**B**) Quantification of TUNEL-positive cells in the heart tissue sections from diabetic rats, expressed as a percentage of total nuclei detected using DAPI staining. Data are presented as means ± SEM (*n* = 4 randomly selected fields per slide, with each slide representing one tissue sample, totaling six samples per group). *** *p* < 0.001 in comparison to normal and diabetic controls, respectively.

The heart tissue sections from the untreated control group exhibited minimal apoptosis, characterized by a low percentage and sparse count of terminal deoxyribonucleotide transferase-mediated dUTP-digoxigenin nick end labeling (TUNEL)-positive cells, resulting in a low score (Figure 5). In contrast, the heart tissue sections from diabetic rats displayed extensive apoptosis, characterized by the highest percentage and count of TUNEL-positive cells among all the groups, correlating with the highest score. Additionally, the heart tissue sections from healthy animals treated with liraglutide demonstrated a subdued apoptotic response, with a low percentage and cell count compared with that in the untreated control group. Conversely, the heart tissue sections from diabetic animals treated with liraglutide exhibited a diminished apoptotic response, with a significantly lower percentage (*p* < 0.001) and count of TUNEL-positive cells, accompanied by a lower overall score (Table S1I,J).

### 2.7. Mitigation of Diabetic Cardiomyopathy by Liraglutide through Restoration of the Expression of PI3K/AKT in the Heart of Diabetic Rats

**Figure 6:**
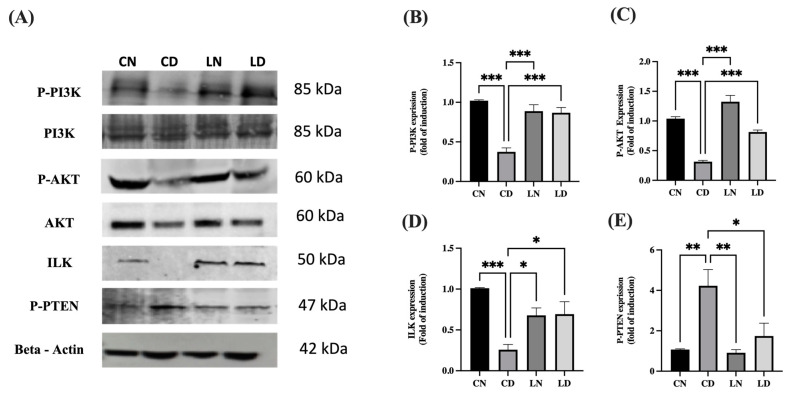
Effect of liraglutide on the levels of integrin-linked kinase (ILK), P-PI3K, P-AKT, and P-PTEN. (**A**) Representative images of Western blots for P-PI3K, P-AKT, ILK, and P-PTEN using the heart tissue lysates. Normal control rats (CN); diabetic controlrats (CD); liraglutide-treated normal rats (LN); and liraglutide-treated diabetic rats (LD). (**B**–**E**) Quantification of bands for P-PI3K (**B**), P-AKT (**C**), ILK (**D**), and P-PTEN (**E**) using ImageJ software. Normalization was performed using total PI3K and AKT for P-PI3K and P-AKT levels, respectively, whereas β-actin levels were used for normalization of ILK and P-PTEN levels. Levels of expression are presented in the form of fold induction relative to normal rats in the control group. Data are exhibited in the form of means ± SEM (*n* = 4 samples from four rats in each group). One-way ANOVA and then the Tukey–Kramer post hoc test was used for statistical analyses and inter-group comparisons. Levels of significance are expressed as ** *p* < 0.01, *** *p* < 0.001 compared with the control group containing normal rats, and * *p* < 0.05, ** *p* < 0.01, *** *p* < 0.001 compared with the control group containing diabetic rats.

As shown in Figure 6A–D and Figure S1, the protein levels of phosphorylated PI3K (*p* < 0.001) (Table S1K), phosphorylated AKT (*p* < 0.001) (Table S1L), and ILK (*p* < 0.05) (Table S1M) in liraglutide-treated diabetic groups were notably increased in comparison to the respective levels in diabetic control groups. The significant reduction in the protein levels of phosphorylated PTEN in the liraglutide-treated diabetic groups compared with those in the diabetic control groups (*p* < 0.05) confirmed the role of PTEN in the negative regulation of PI3K and overexpression of ILK (Figure 6E) (Table S1N).

## 3. Discussion

This study was aimed at obtaining conclusive evidence for the potential of liraglutide in mitigating diabetic cardiomyopathy via the modulation of the ILK/PI3K/Akt/PTEN signaling pathways. A comprehensive understanding of these pathways and their interplay with GLP1R is pivotal for identifying novel therapeutic targets and for devising strategies with potential clinical applications in diverse cardiovascular diseases, particularly DCM. DCM, an unfavorable condition resulting from type 2 diabetes [31], was modeled in this study using the STZ/high-fat diet (HFD) model [32]. This model is renowned for inducing characteristics akin to type 2 diabetes, including insulin resistance, which affects glucose uptake by cardiac cells and disrupts energy metabolism, thereby leading to cardiac anomalies resembling diabetic cardiomyopathy [33,34]. The early stages of DCM manifest various pathological changes, including left ventricular hypertrophy, cardiac fibrosis, inflammation, and oxidative stress [35,36,37].

Both the heart weight-to-body weight ratio and fasting blood glucose levels were improved in rats when they were treated with liraglutide, thus mitigating cardiovascular risk factors [38]. These findings align with our results, corroborating the protective effect of liraglutide against DCM reported in a previous study [27]. A significant reduction in the heart weight-to-body weight ratio observed in rats with diabetes that received liraglutide treatment—a pattern commonly observed in preclinical studies and clinical trials—further supports the cardioprotective effects of liraglutide [39,40,41].

Validation of the DCM model involved measurement of the cardiac biomarkers, troponin I, and CK-MB. The elevation of these biomarkers in STZ-treated and HFD-fed rats validates the model, and the mitigation of these cardiac biomarkers by liraglutide underscores its cardioprotective effect, as evidenced in the histopathological examination. Oxidative stress, a critical aspect in the process by which type 2 diabetes develops [42], manifests when there is a lack of balance between the generation and elimination of reactive oxygen species (ROS). This imbalance leads to excessive accumulation of ROS, resulting in damage to tissues, cells, and biological macromolecules, including nucleic acids and proteins. Oxidative stress has been implicated in functional injury to islet β cells and peripheral insulin resistance, contributing to the onset of diabetes and its severe complications, including cardiovascular diseases [43,44]. In the existing study, liraglutide treatment attenuated apoptosis of heart cells in diabetic rats, underscoring the substantial contribution of antiapoptotic effects to its cardioprotective action against DCM.

Apoptosis, a pivotal factor in DCM and a manifestation of ischemic myocardial injury [45,46], is more pronounced in diabetic rats than in nondiabetic rats during ischemia/reperfusion-induced apoptotic injuries. Myocardial cell injury due to apoptosis has been identified as a significant cause of various heart diseases [45,47]. In this study, as a result of being treated with liraglutide, diabetic rats exhibited significantly reduced levels of BAX and caspase 3 expression. It has been highlighted that when the PI3K/AKT signaling pathway is activated, this facilitates a reduction in apoptosis and regulation of the processes by which glucose is transported and glycogen is synthesized [48]. Specifically, AKT exhibits a cytoprotective function through the activation of downstream effector molecules, including the antiapoptotic BCL2 [46,49]. The improvement in cardiovascular function in diabetic patients by liraglutide and its protection against DCM is attributed, in part, to its inhibition of the endoplasmic reticulum (ER) stress pathway, which occurs independent of its glucose-lowering effect. It is pertinent to mention that ER is a critical factor in the intrinsic apoptosis pathway [50].

Chronic hyperglycemia is implicated in direct impairment of the PI3K/AKT signaling pathway, which is a pivotal step in insulin action [51]. The PI3K/AKT signaling pathway, known for its effects on cardiomyocyte survival and function, is activated by insulin, thereby promoting cell survival [10,52]. Regarding ILK, hyperglycemia and hyperinsulinemia were observed in ILK-depleted mice [53]. Furthermore, low levels of cardiac ILK have been observed in an animal model of diabetic cardiomyopathy [54]. Consistently, in this study, the STZ-induced type 2 diabetes model showed a significant decrease in cardiac ILK expression, indicating that diabetes affects ILK expression in the heart. Interestingly, cardiac ILK depletion in mice reportedly promotes insulin resistance by increasing the phosphorylation of insulin receptors and decreasing the phosphorylation of AKT in conjunction with decreased GLUT4 transcription [53]. This is consistent with our findings, as we observed that decreased ILK expression in the cardiac tissue of diabetic rats was accompanied by decreased phosphorylation of PI3K and AKT. Conversely, the present study revealed a noteworthy increase in the levels of ILK, phosphorylated PI3K, and phosphorylated AKT in diabetic rats that were administered the liraglutide treatment compared with those in the diabetic control group. Additionally, there was a substantial reduction in the levels of phosphorylated PTEN in diabetic rats that received liraglutide treatment compared with those in the diabetic control group. This underscores the significance of ILK not only as a kinase that influences PI3K/AKT but also as a regulator of PTEN, affirming the role of PTEN as a negative regulator of PI3K and ILK overexpression [12,55]. Thus, this study suggests that the cardioprotective effect of liraglutide against DCM could be exerted via modulation of the ILK/PI3K/AKT/PTEN signaling pathway. Previous reports have indicated that GLP-1 receptors are expressed within cardiomyocytes [56]. Real-time quantitative polymerase chain reaction and in situ hybridization studies have detected GLP1R mRNA transcripts and the GLP-1R immunoreactive protein within cardiomyocytes of human tissue [57]. Further, these findings were validated using available single-cell RNA sequencing datasets from human and mice tissues [58]. In line with this, the genetic deletion of GLP-1 in mice (*Glp1r*^−/−^) resulted in heart malformation and dysfunction [59]. Additionally, the GLP-1 receptor is expressed in the pancreas. Thus, it may indirectly modulate the heart’s functional output via regulating endocrine and physiological functions, including insulin, fatty acids, glucose, and glucagon metabolism [60].

Accumulating evidence has shown that targeting GLP-1 signaling is an effective approach to improving cardiovascular outcomes in preclinical models of diabetes [61]. Our findings of liraglutide’s cardioprotective role through modulation of oxidative stress align with previous findings [60]. The pharmacological targeting of GLP-1 receptors has been shown to reduce inflammatory responses via the Akt protein kinase pathway [62]. Furthermore, the pharmacological modulation of phosphoinositide 3-kinase (PI3K) by liraglutide has been supported previously [60]. Also, the molecular modulation of P13K/Akt/PTEN signaling in the streptozotocin-induced diabetic model is already established [63]. It was reported that the pharmacological application of a GLP-1 agonist modulates the ILK in human HepG2 cells [64]. Physiologically, Integrin-Linked Kinase is linked to the modulation of glucoregulation [65]. Further, a mutant glioblastoma cell line lacking the PTEN gene was associated with the activation of PI3K-dependent signaling and suppression of the ILK pathway [12]. This evidence supports the rationale of our central hypothesis and our findings that liraglutide weakens cardiomyopathy streptozotocin-induced diabetes through the modulation of the ILK and PI3K/AKT/PTEN pathway.

It is worth noting that the concept of liraglutide attenuating diabetic cardiomyopathy is well established. Diabetic complications are complex and prominent diseases, and multiple pathological mechanisms, including oxidative stress [36], apoptosis, inflammation, and mitochondrial dysfunction [66], are linked. Among molecular pathways, the PI3K/Akt/PTEN signaling stands out, especially in the context of diabetes nephropathy [63]. The modulation of PI3K/Akt/PTEN by liraglutide has been examined previously but in the context of hepatocellular carcinoma [67]. However, studies directly mapping PI3K/Akt/PTEN in diabetic cardiomyopathy following liraglutide treatment are sparse. Additionally, ILK is functionally associated with glucoregulation [65], yet it has not been examined in this context. As far as we know, no previous report has examined the molecular alterations of ILK and PI3K/AKT/PTEN pathways in this context. Through this work, we highlight potential molecular targets in diabetic cardiomyopathy.

In summary, our findings indicate that liraglutide significantly ameliorates hyperglycemia, cardiac injury, and oxidative stress biomarkers in rats with STZ-induced type 2 diabetes, confirming its capacity to shield the heart from the deleterious effects of hyperglycemia. Liraglutide effectively restored the compromised ILK/PI3K/AKT signaling pathway in the heart of diabetic rats by elevating the levels of ILK, phosphorylated PI3K, and AKT while concurrently reducing PTEN phosphorylation. Thus, liraglutide demonstrated a positive effect on diabetic cardiomyopathy in rats, potentially through the ILK-associated PI3K/AKT/PTEN axis. Despite the limited evidence highlighting the significance of targeting GLP1R as a potential treatment for DCM, to our knowledge, no study has yet elucidated the interplay between GLP1R and the ILK/PI3K/AKT/PTEN signaling pathways in the context of DCM.

### Study Limitations

This study had minor limitations that did not drastically affect the study findings. Most arose because hemodynamic and echocardiographic parameter assessments were not included in the study. However, excluding these assessments was justified because this study was intended to be a mechanistic study to investigate molecular targets that drive liraglutide-attenuated diabetic cardiomyopathy. Nevertheless, in future studies with a main focus on the clinical presentation of liraglutide effects, the assessment of these parameters will be crucial. In the present study, we conducted a considerable number of experiments, including cardiac injury biomarker assessment, heart weight-to-body weight ratio measurements (as an index of heart hypertrophy), and histological examinations, to confirm the cardioprotective effect of liraglutide. We recognize that many factors related to cardiac tissue preparation and cardiac tissue complexity, as well as technical issues related to image processing software, can decrease the quality of cardiac tissue images. Therefore, future studies should include the use of cardiac cell lines or isolated primary cardiomyocytes and highly sophisticated imaging techniques as alternative options to optimize the experimental conditions and improve the quality of cardiac images.

## 4. Materials and Methods

### 4.1. Drugs, Chemicals, and Antibodies

STZ was sourced from Sigma-Aldrich (St. Louis, MO, USA), and liraglutide (Victoza) was procured from Merck & Co., Inc. (Whitehouse, NJ, USA). The apoptotic biomarker, caspase 3, was assessed using a specific ELISA kit designed for rats from Abcam Biotechnology Inc. (Cambridge, UK). The TUNEL assay kit (In Situ Cell Death Detection Kit)-HRP-diaminobenzidine (DAB) was procured from Abcam Biotechnology Inc.

For quantitation of cardiac biomarkers (troponin I and CK-MB), ELISA kits specifically designed for rats were obtained from Cloud-Clone Corp. (Houston, TX, USA). Santa Cruz Biotechnology, Inc. (Dallas, TX, USA) was contacted to acquire caspase 3 monoclonal antibody. β-Actin, employed as a housekeeping loading control antibody, in addition to different primary antibodies with relevance (ILK, BAX, Bcl2, P-PTEN, P-AKT/AKT, and P-PI3K/PI3K), was bought from Abcam. Secondary anti-goat, anti-mouse, and anti-rabbit antibodies were purchased from Sigma-Aldrich. All other chemicals and reagents were suitable for analytical purposes and were sourced from conventional commercial firms.

### 4.2. Experimental Animals

All rats were male Wistar albino models, with ages ranging from 8 to 10 weeks and a weight of 200 to 250 g; they were acquired from the Animal Care Center at the College of Pharmacy, King Saud University, Saudi Arabia. All rats were housed and handled appropriately and were stored in cages designed for the specific purpose in a controlled environment with a standard temperature (22–23 °C), relative humidity (60%), and a 12/12 h light/dark cycle. Throughout the experiment, rats were fed rodent maintenance chow and had ad libitum access to distilled water. A regular diet was given to rats in the control group, whereas an HFD was given to control group rats used for induction of insulin resistance and obesity. The HFD consisted of a mixed meal containing 63% calories, 1% (*w*/*w*) sucrose, 1% (*w*/*w*) cholesterol, elevated protein levels, and 25% extra virgin olive oil. It was ensured that the study protocol conformed with the guidelines published by the Experimental Animals Ethics Committee Acts of King Saud University Institutional Animal Care and Use Committee, and approval was obtained from the Research Ethics Committee (reference No. KSU-SE-21-40). Preliminary research performed in various laboratories was used as the basis for the regimen adopted in the study.

### 4.3. Induction of Diabetes

To induce type 2 diabetes in rats, STZ (30 mg/kg) was injected intraperitoneally once after 24 h fasting and was aided by feeding an HFD [68,69]. Immediately before being injected, STZ was dissolved in 0.1 M of citrate buffer (pH 4.5). Seventy-two hours later, a small sample of blood was taken from the vein in the tail for the purpose of assessing the levels of blood glucose using an Accu-Chek Advantage II blood glucose monitoring system (Roche Diagnostics, Indianapolis, IN, USA). Blood glucose levels > 200 mg/dL in two consecutive assessments indicated diabetes in rats [70]. The rats with diabetes were chosen for experiments and were assigned to groups 3 and 4, as described in Section 2.4.

### 4.4. Experimental Design

**Figure 7:**
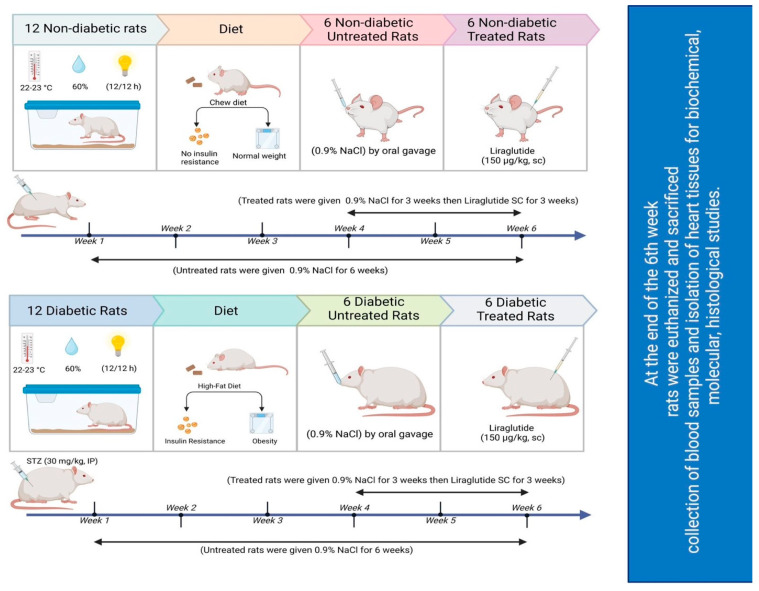
Schematic of the study design.

The experimental design and approach used in this study are illustrated in Figure 7. Twenty-four rats were divided into 2 groups of 12 nondiabetic and 12 diabetic rats, which were weighed and subsequently allocated to 4 groups of six rats each (*n* = 6). Over the course of six consecutive weeks, the rats were subjected to the following daily treatments:

- Group 1: Normal saline (0.9% NaCl; drug vehicle) was administered to nondiabetic controls via oral gavage for the entire six-week period.
- Group 2: Normal saline (0.9% NaCl) was administered to diabetic untreated rats via oral gavage for six weeks.
- Group 3: Normal saline was administered to nondiabetic rats for the initial 3 weeks before they were injected with 150 μg/kg liraglutide subcutaneously (SC) twice daily for the subsequent 3 weeks. The dose was chosen based on previous studies demonstrating its cardioprotective effects in diabetic rats [71]. Notably, the volume of liraglutide stock solution (6 mg/mL) injected was dependent on each rat’s weight.
- Group 4: Normal saline was administered to diabetic rats for the initial 3 weeks, and they were subsequently injected with 150 μg/kg liraglutide SC twice daily [71] for the remaining 3 weeks.

Weekly body weight records were maintained until the end of the experiment. Following 12 h overnight fasting, the rats were humanely euthanized by gradually increasing the carbon dioxide concentration, followed by decapitation. Thereafter, blood samples were collected, and the serum was separated for subsequent biochemical analyses. The heart was promptly excised after the sacrifice, rinsed with cold phosphate-buffered saline (PBS), and weighed. The heart weight-to-body weight ratio (HW/BW) was calculated as an indicator of cardiac hypertrophy and injury. The heart samples were homogenized in cold PBS (10% *w*/*v*), and the homogenate was used to assess oxidative stress biomarkers via specific ELISA and commercial kits. Serum caspase 3 levels were determined using ELISA kits that were specifically designed for rats. Subsequent to dissection, the heart tissue was preserved in neutral buffered formalin (4%) for subsequent histological analysis, apoptotic cardiomyocyte staining, and immunohistochemical processing. Samples were also stored at −80 °C for further use in molecular studies.

### 4.5. Biochemical and Molecular Analyses

#### 4.5.1. Determination of Serum Glucose Levels

A glucose analytical assay was used to determine the serum glucose levels according to the instructions provided by manufacturer.

#### 4.5.2. Determination of Diabetic Cardiomyopathy Biomarkers

ELISA kits were used on serum samples to determine the serum levels of cardiac biomarkers, which included troponin I and CK-MB, in line with the manufacturer’s instructions. For the troponin I and CK-MB assays, 50 μL of each standard or sample was incubated in the wells of ELISA plates at 37 °C for 1 h. Subsequently, the wells were washed to eliminate all unbound substances. Following the addition of 50 μL of the selected primary antibody to all wells, incubation of the plates was performed at 37 °C for 1 h. Subsequently, 50 μL of a designed secondary antibody was introduced to all wells, followed by incubation of the plates at 37 °C for 1 h. After this had been completed, this was followed by the addition of 100 μL of the substrate to all wells and then a further 30 min of incubation at 37 °C in a dark environment. The final part of process involved the addition of 100 μL of the stop solution to every well, and then a microplate reader (BioTek Instruments, Winooski, VT, USA) was used to measure the absorbance at 450 nm. The calculation of the sample concentrations was performed with a standard curve created according to the guidelines provided with the assay kit.

#### 4.5.3. Estimation of Oxidative Stress Biomarkers

Oxidative stress was assessed by measuring the activities of SOD and GPx and MDA levels. In order to determine GPx activity, the method proposed by Moron et al. was used [72]. In summary, this involved the mixing of 1 mL of the heart homogenate and 1 mL of 25% trichloroacetic acid (TCA), followed by the centrifugation of the resulting mixture at 3000 rpm for 10 min at 4 °C. This was followed by the mixing of 0.5 mL of the supernatant with 4.5 mL of Ellman’s reagent, and then spectrophotometric measurements of the yellow mixture’s absorbance at 412 nm against a reagent blank were taken.

MDA levels were estimated with a thiobarbituric acid (TBA) assay according to the description of Ohkawa et al. [73]. This involved the heating of a mixture that contained 1 mL of 0.6% TBA, 2.5 mL of 20% TCA, and 500 μL of the heart homogenate over a bath of boiling water bath for 30 min, and it was then cooled and centrifuged at 4 °C. Measurements of the color that was formed were taken at 535 nm against a reagent blank. The nitro blue tetrazolium technique was used to determine the SOD activity, as described by Delides et al. [74]. This method determines the extent to which the reduction of nitro blue tetrazolium is inhibited by measuring the change in absorbance at 430 nm. The activities of SOD and GPx are denoted as u/mg protein, while MDA content is denoted as nmol/mg protein.

#### 4.5.4. Western Blot Analysis

Homogenization of samples of frozen heart tissue was performed in an ice-cold lysis buffer followed by a radioimmunoprecipitation assay buffer, in which identical concentrations of protease and phosphatase inhibitor cocktails were supplemented. The Direct Detect qualification assay method was used to determine the protein concentrations. Western blot analysis was performed using the technique described by Towbin et al. [75]. In brief, sodium dodecyl sulfate polyacrylamide gel electrophoresis was used to separate 60 μg of homogenized protein samples, followed by transferal of the resolved proteins into polyvinylidene difluoride membranes (0.2 μm, Immun-Blot^®^, Bio-Rad, Hercules, CA, USA). Blocking of the membrane was achieved via a process in which it was incubated in a 5% non-fat dry milk solution at room temperature for 1 h. Following this, overnight incubation of the membrane was performed at 4 °C where primary antibodies were diluted in Tris-buffered saline containing Tween-20 (TBST) buffer at a 1:1000 ratio for caspase 3, BAX, BCL2, ILK, P-PTEN, P-PI3K/total PI3K, and P-AKT/total AKT. A mouse monoclonal anti-β-actin antibody, diluted at 1:2000, was used to assess the levels of β-actin as a loading control. This was followed by washing and incubation of the membrane at room temperature for 1 h with HRP-conjugated anti-rabbit (1:5000) secondary antibody diluted in the TBST buffer. An enhanced chemiluminescence detection kit (GE Healthcare, Amersham, Buckinghamshire, UK) was used to develop the blots for 2 min. A LI-COR Odyssey imaging system (Lincoln, NE, USA) was used to visualize the immunoreactive bands. ImageJ software was used to densometrically quantify the protein band intensities (NIH Image, Bethesda, MD, USA), which were then normalized relative to the loading control intensity (β-actin) or the intensity of total protein in case of detection of the expression of phosphorylated proteins. This was followed by the normalization of the relative values against those for the control, which were allocated an arbitrary value of 1 and denoted as fold change.

#### 4.5.5. Histological Examination

The rats were anesthetized with carbon dioxide, and their hearts were promptly removed, washed with ice-cold saline, and meticulously cleaned of connective tissue and extraneous fat. Preservation of the hearts in 10% neutral formalin for 24 h was performed immediately. Once this had been completed, it was followed by dehydration of the hearts in elevated concentrations of ethanol, clearing with xylene, and they were finally embedded in paraffin. In preparation for morphological examination, sections of the paraffin-embedded hearts (thickness 4 μm) were stained with H&E. A ScanScope Scanner (Leica Biosystems, Aperio, Vista, CA, USA) was used to obtain high-resolution digital scans of the stained sections. Expert investigators with experience in the field then analyzed the images.

#### 4.5.6. Immunohistochemistry

Immunohistochemistry was performed using a kit from Advanced Technology & Industrial Co., Ltd. (Hong Kong, China) to measure ILK expression, following the manufacturer’s instructions. Paraffin-embedded heart sections were used to detect the expression of caspase 3, BAX, BCL2, ILK, PTEN, P-PI3K, and P-AKT. Immunostaining was performed as previously described using the Santa Cruz ImmunoCruz ABC staining system. To prevent the non-specific binding of antibodies, incubation of the protein sections was performed for 5 min. This was followed by the overnight incubation of the sections with the primary antibodies at 4 °C and subsequent washing using Tris buffer three times. Then, incubation of the sections with anti-mouse IgG or biotinylated anti-rabbit (both at a 1:100 dilution) was performed for 30 min. Washing of the sections was then repeated, followed by staining using a working solution of DAB, then staining using Mayer’s hematoxylin. Subsequent to staining, preservation of the sections was achieved by mounting them in a mixture containing xylene and a plasticizer. A bright-field light microscope (DMRBE; Leica, Bensheim, Germany) that had a video camera installed (ProgRes; Kontron Instruments, Watford, UK) was used to analyze the samples. Aperio viewing and image analysis tools were subsequently used to view and analyze the digital images of the sections.

#### 4.5.7. Detection of Apoptotic Cardiomyocytes

Apoptosis of cardiomyocytes was detected using the TUNEL staining assay, following the manufacturer’s instructions. Proteinase K (20 μg/mL) was used to treat deparaffinized heart tissue slides for 15 min at room temperature, and they were then quenched in 2% hydrogen peroxide. Subsequent to being rinsed with PBS (pH 7.4), incubation of the slides in 1× equilibration buffer was performed for 10–15 s. They were then incubated with terminal deoxynucleotidyl transferase for 1 h at 37 °C, blocked using a stop/wash buffer, then incubated with peroxidase-conjugated anti-digoxigenin antibody for 30 min at room temperature. DAB (Sigma-Aldrich) was used to develop the slides. Finally, the tissue sections stained with fluorescently tagged dUTP were visualized using a Nikon E100 Binocular Fluorescence Microscope (Nikon Corporation, Tokyo, Japan). Nuclear staining was performed with 4′,6-diamidino-2-phenylindole (DAPI) as a counterstain. To calculate the cardiomyocyte apoptosis index, the number of apoptotic cardiomyocytes was divided by the overall number of cardiomyocytes. This value was multiplied by 100 [76]. All analytical procedures were performed in a blinded manner.

### 4.6. Statistical Analysis

Data are presented in the form of means ± SEM. One-way analysis of variations and then the Tukey–Kramer post hoc test was used for statistical analysis of inter-group comparisons. All statistical analyses were conducted using GraphPad Prism Windows software version 9 (GraphPad Software Inc., San Diego, CA, USA). *p* values < 0.05 indicated statistical significance.

## 5. Conclusions

Our findings suggest that liraglutide, through its targeting of GLP1Rs, affects the expression of ILK-related PI3K/AKT/PTEN, ultimately mitigating diabetic cardiomyopathy by regulating these signaling pathways. Thus, liraglutide is a potential alternative treatment for treating diabetic cardiomyopathy.

## Figures and Tables

**Table 1:** Effect of liraglutide on body weight, heart weight-to-body weight ratio, and blood glucose levels in rats.

Groups	Parameters		
	Body Weight (g)	HW/BW Ratio (mg/g)	Glucose (mg/dL)
Nondiabetic control	389.2 ± 17.76	2.619 ± 0.27	39.37 ± 6.22
Diabetic control	252.0 ± 13.16 ^###^	4.751 ± 0.49 ^###^	323.2 ± 27.64 ^###^
Liraglutide-treated nondiabetic rats	340.8 ± 10.20	2.774 ± 0.1	37.83 ± 5.82
Liraglutide-treated diabetic rats	220.0 ± 13.20	2.798 ± 0.13 ***	62.38 ± 3.83 ***

Data are expressed in the form of means ± standard error of the mean (SEM; *n* = 6 samples per group). One-way analysis of variance (ANOVA) was performed for comparison of data, followed by the Tukey–Kramer post hoc test. We first compared diabetic control group with the normal (nondiabetic) control group (^###^ *p* < 0.001) and applied the same process for liraglutide-treated diabetic and liraglutide-treated nondiabetic groups (*** *p* < 0.001). Abbreviations: HW/BW, heart weight-to-body weight.

## Data Availability

The study data are available upon request from the corresponding author.

## Supplementary Materials

The following supporting information can be downloaded at: https://www.mdpi.com/article/10.3390/ph17030374/s1, Figure S1: Original unprocessed immunoblots for all proteins of interest. Table S1: Original data for all biochemical and molecular parameters.
