# Supplementary material for: Liraglutide Attenuates Diabetic Cardiomyopathy via the ILK/PI3K/AKT/PTEN Signaling Pathway in Rats with Streptozotocin-Induced Type 2 Diabetes Mellitus
Source: Pharmaceuticals (Basel). 2024 Mar 15;17(3):374. doi: 10.3390/ph17030374 (PMC10975938; doi:10.3390/ph17030374)
Supplement: Supplementary file 1 [file pharmaceuticals-17-00374-s001.zip › pharmaceuticals-2911073-Figure S1.pdf.pdf]

**Figure S1. Original unprocessed immunoblots for all all proteins of interest**

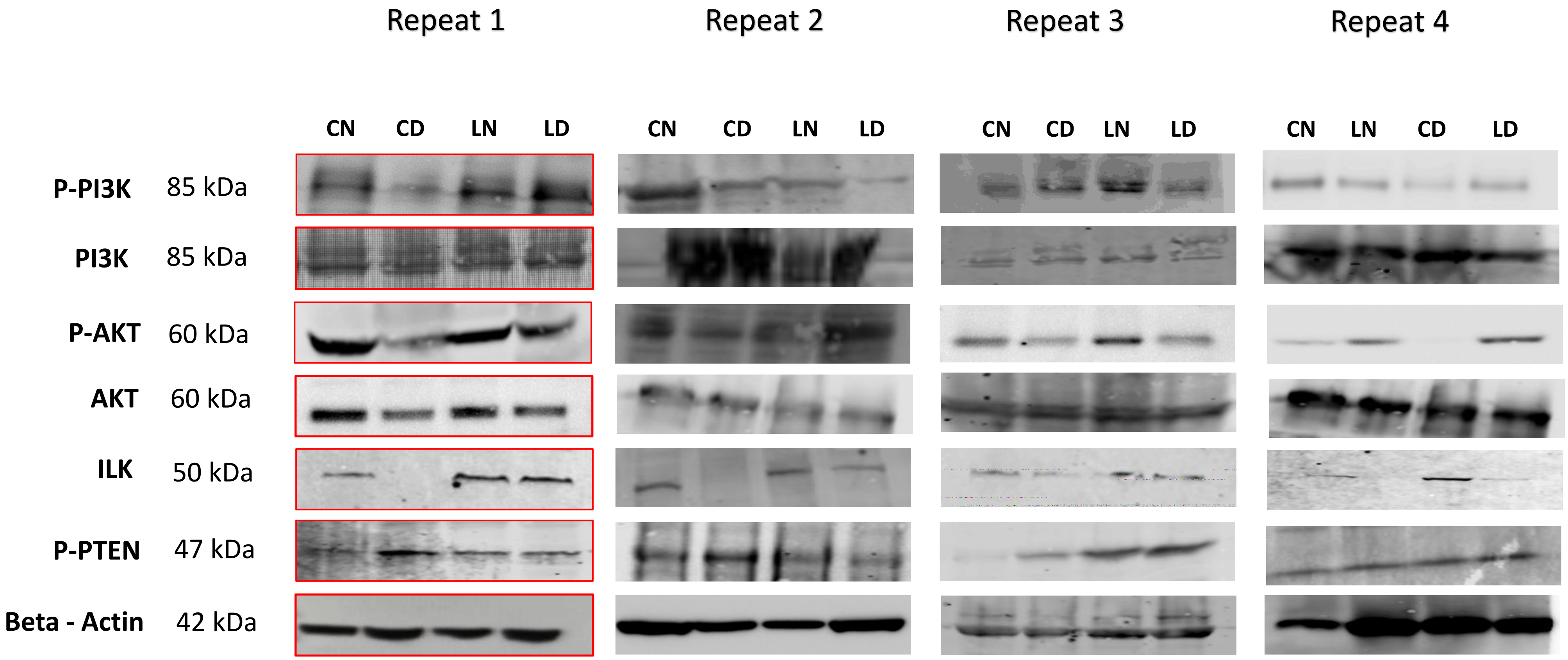

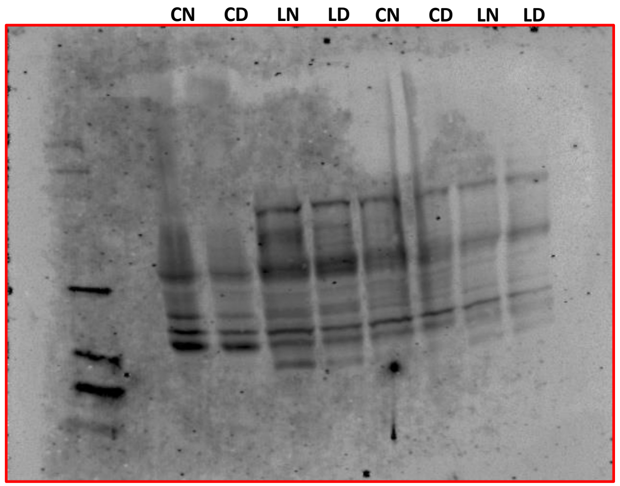
CN CD LN LD CN CD LN LD

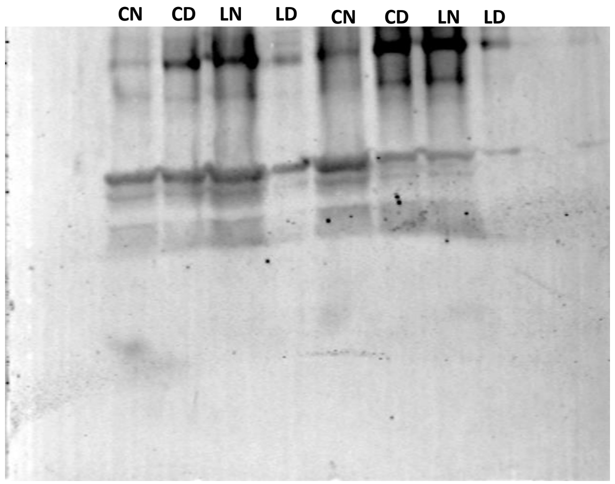
CN CD LN LD CN CD LN LD

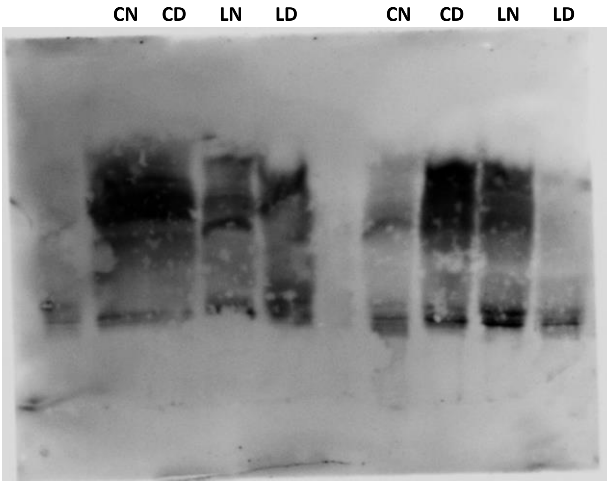
CN CD LN LD CN CD LN LD

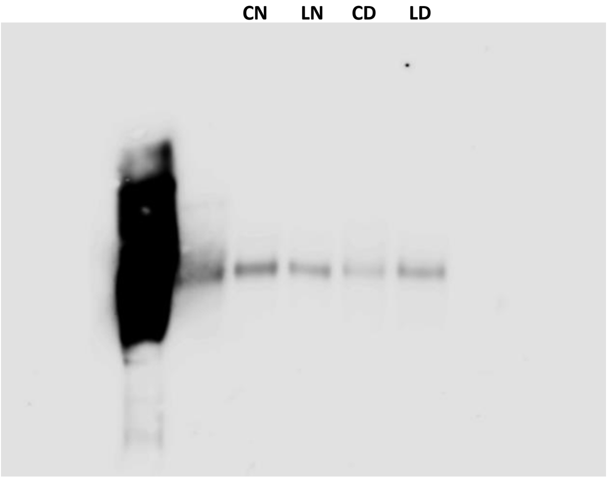
CN LN CD LD

P-PI3K

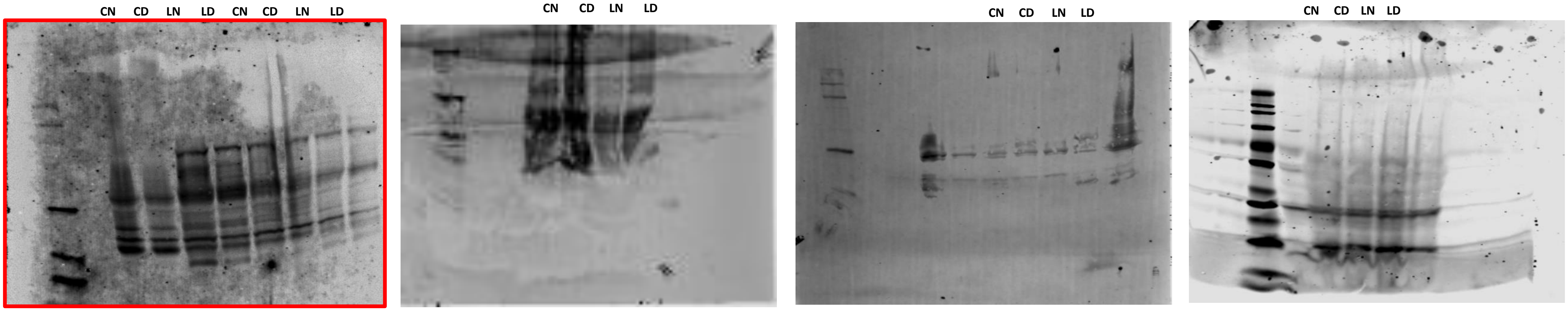


PI3K

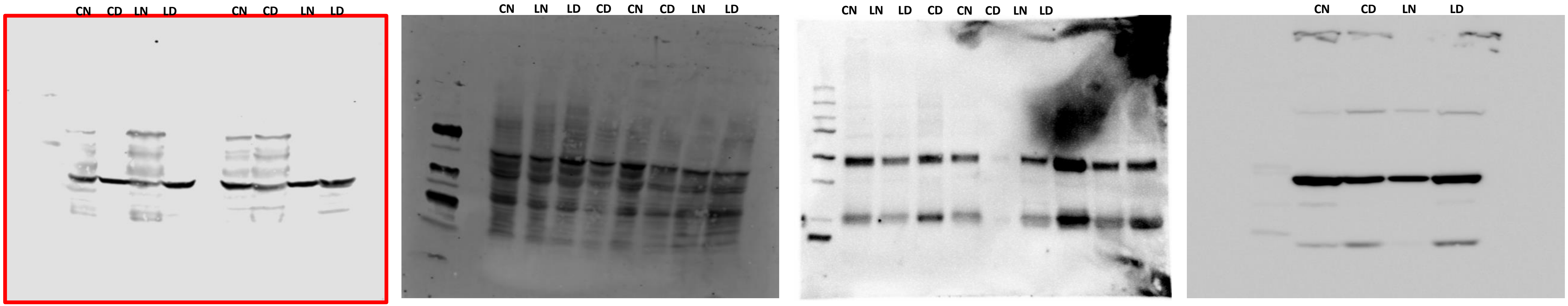
CN CD LN LD CN CD LN LD
CN LN LD CD CN CD LN LD
CN LN LD CD CN CD LN LD
CN CD LN LD

P-AKT

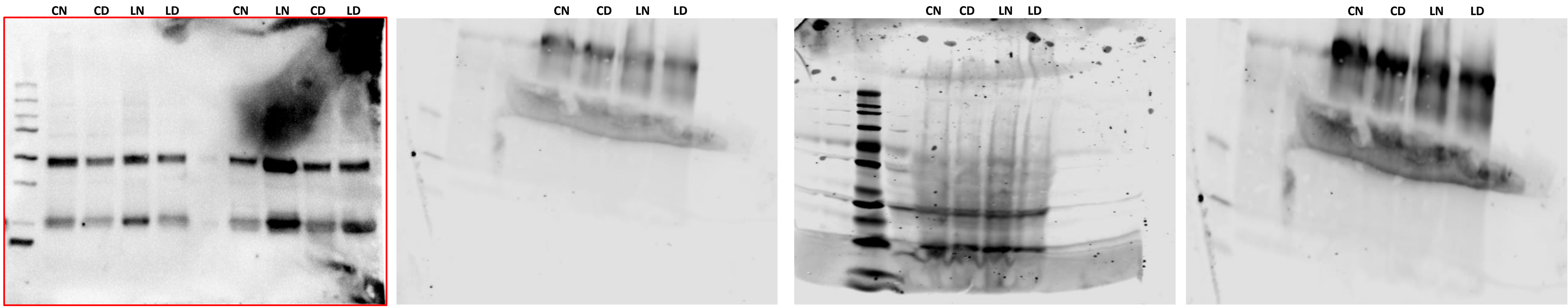


AKT

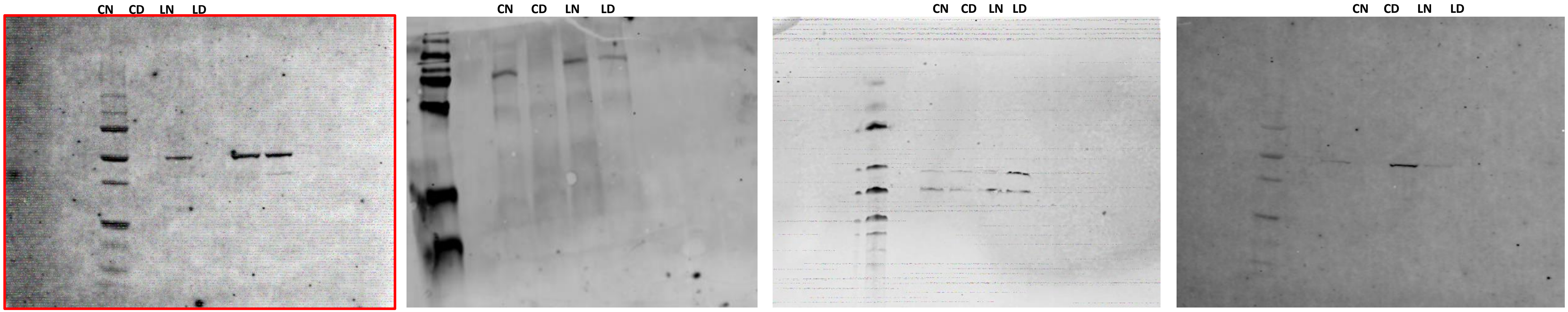


ILK

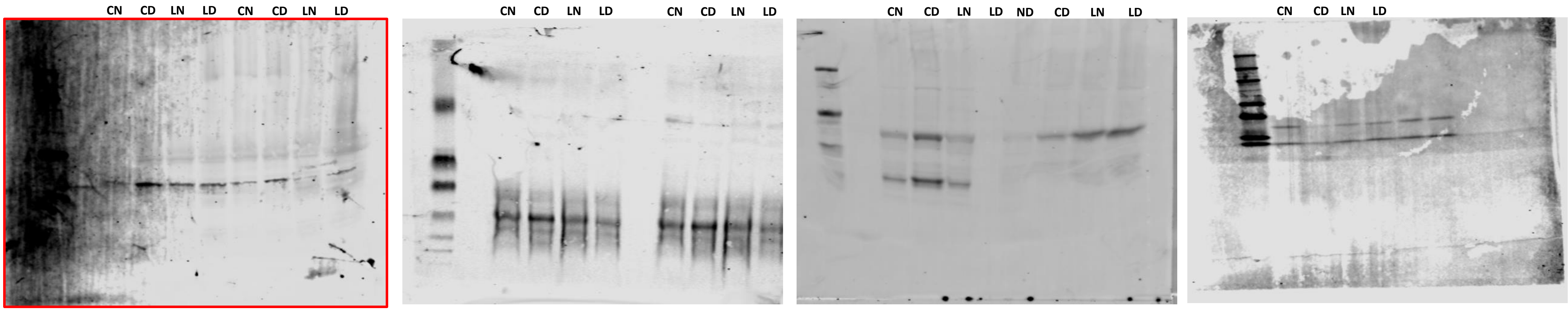
CN CD LN LD CN CD LN LD
CN CD LN LD CN CD LN LD
CN CD LN LD ND CD LN LD
CN CD LN LD

P-PTEN

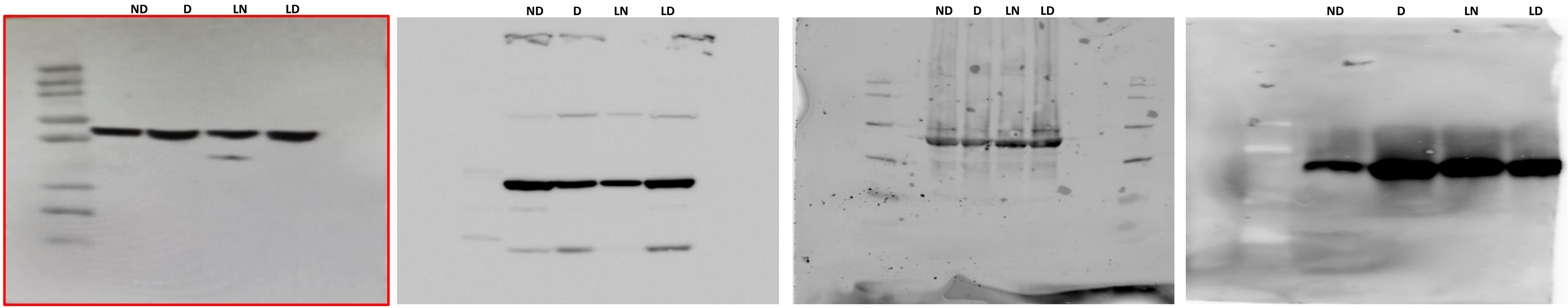


$\beta$-Actin
