# Supplementary material for: Liraglutide Attenuates Diabetic Cardiomyopathy via the ILK/PI3K/AKT/PTEN Signaling Pathway in Rats with Streptozotocin-Induced Type 2 Diabetes Mellitus
Source: Pharmaceuticals (Basel). 2024 Mar 15;17(3):374. doi: 10.3390/ph17030374 (PMC10975938; doi:10.3390/ph17030374)
Supplement: Supplementary file 1 [file pharmaceuticals-17-00374-s001.zip › pharmaceuticals-2911073-Table S1.pdf]

Table S1 (A). Original data for the effects of liraglutide on body weight (g) in diabetic rats.

	Non-diabetic control	Diabetic control	Liraglutide-treated non-diabetic rats	Liraglutide-treated diabetic rats
Repeat no. 1	365	245	307	275
Repeat no. 2	352	292	377	322
Repeat no. 3	397	257	352	334
Repeat no. 4	366	240	354	361
Repeat no. 5	472	200	325	339
Repeat no. 6	383	278	330	289

Table S1 (B). Original data for the effects of liraglutide on heart weight/body weight ratio (mg/g) in diabetic rats.

	Non-diabetic control	Diabetic control	Liraglutide-treated non-diabetic rats	Liraglutide-treated diabetic rats
Repeat no. 1	3.20547945	5.30612245	2.50814332	2.90909091
Repeat no. 2	3.23863636	3.97260274	3.02387268	2.73291925
Repeat no. 3	1.76322418	3.19066148	2.5000000	2.2754491
Repeat no. 4	1.83060109	4.08333333	2.96610169	2.90858726
Repeat no. 5	2.75423729	6.45000000	2.76923077	2.74336283
Repeat no. 6	2.92428198	5.50359712	2.87878788	3.21799308

Table S1 (C). Original data for the effects of liraglutide on glucose levels (mg/dl) in diabetic rats.

	Non-diabetic control	Diabetic control	Liraglutide-treated non-diabetic rats	Liraglutide-treated diabetic rats
Repeat no. 1	20.6395349	119.767442	86.0465116	62.6453488
Repeat no. 2	65.6976744	120.784884	82.2674419	64.8255814
Repeat no. 3	32.4127907	114.97093	47.8197674	46.5116279
Repeat no. 4	32.994186	124.273256	62.7906977	67.005814
Repeat no. 5	39.9709302	103.488372	60.755814	74.5639535
Repeat no. 6	44.4767442	75.5813953	67.2965116	58.7209302

Table S1 (D). Original data for the effects of liraglutide on serum cardiac biomarkers (Troponin-I (pg/mL)) in diabetic rats.

	Non-diabetic control	Diabetic control	Liraglutide-treated non-diabetic rats	Liraglutide-treated diabetic rats
Repeat no. 1	132.94894630658	261.268043036461	158.81684083682	198.864810441775
Repeat no. 2	139.996013919469	342.298418318752	158.81684083682	177.207968509482
Repeat no. 3	125.472179449003	223.787366633701	155.207367181438	196.028795426832
Repeat no. 4	98.315187184698	242.436313853176	132.94894630658	177.981427149921
Repeat no. 5	122.808044131935	284.289020285822	116.190675763734	159.676239326197
Repeat no. 6	116.792254706298	224.732704972015	120.745487757431	113.956239691355

Table S1 (E). Original data for the effects of liraglutide on serum cardiac biomarkers (CK-MB (ng/mL)) in diabetic rats.

	Non-diabetic control	Diabetic control	Liraglutide-treated non-diabetic rats	Liraglutide-treated diabetic rats
Repeat no. 1	6.33618197145725	29.9750147111247	13.1597625561035	5.91841173158095
Repeat no. 2	3.23771935904134	19.6700154608426	6.23173941148818	17.1982082082411
Repeat no. 3	3.72511797223036	33.7001326833551	13.7167895426053	11.2797964766602
Repeat no. 4	8.35540479752605	28.2343053783068	4.2821449587321	3.02883423910319
Repeat no. 5	6.5450670913954	19.809272207468	13.4382760493544	3.44660447897949
Repeat no. 6	12.0457085831001	14.0301172225125	28.0254202583686	8.98206015734051

Table S1 (F). Original data for the effects of liraglutide on cardiac oxidative stress markers (MDA (mmol/g)) in diabetic rats.

	Non-diabetic control	Diabetic control	Liraglutide-treated non-diabetic rats	Liraglutide-treated diabetic rats
Repeat no. 1	39.5	92.75	10.25	26.25
Repeat no. 2	13	18.5	9	26.75
Repeat no. 3	27	79.25	14.5	56
Repeat no. 4	32.5	44.5	13.5	46.75
Repeat no. 5	16.25	68.75	11.75	16.5
Repeat no. 6	39.75	72.5	9.25	21.25

Table S1 (G). Original data for the effects of liraglutide on cardiac oxidative stress markers (SOD (U/ml)) in diabetic rats.

	Non-diabetic control	Diabetic control	Liraglutide-treated non-diabetic rats	Liraglutide-treated diabetic rats
Repeat no. 1	1.559	0.873	1.281	1.377
Repeat no. 2	1.805	1.31	1.555	1.488
Repeat no. 3	1.255	0.224	1.545	1.594
Repeat no. 4	0.982	0.124	1.338	1.794
Repeat no. 5	1.838	0.876	1.642	2.052
Repeat no. 6	1.559	0.873	1.281	1.377

Table S1 (H). Original data for the effects of liraglutide on of on cardiac oxidative stress markers (GPX (nmol/min/mg protein)) in diabetic rats.

	Non-diabetic control	Diabetic control	Liraglutide-treated non-diabetic rats	Liraglutide-treated diabetic rats
Repeat no. 1	165.09021088337	84.7988104921902	121.775654580474	86.5345213188017
Repeat no. 2	108.589490959585	99.9835187932504	139.272740716469	115.119545432771
Repeat no. 3	150.624388603053	66.3518509213321	104.751313372528	118.03064939311
Repeat no. 4	171.288354016107	85.8877571097292	91.1317035122362	122.045021647597
Repeat no. 5	157.702719164588	57.0476027172991	102.844376658592	85.4538398984304

Repeat no. 6	123.900853501179	55.551943776153	102.139010126065	125.833234299624

Table S1 (I). Original data for the effects of liraglutide on diabetic cardiac tissue apoptosis showing the numbers of Tunel-postive cells in all experimental groups versus controls.

	Non-diabetic control	Diabetic control	Liraglutide-treated non-diabetic rats	Liraglutide-treated diabetic rats
Repeat no. 1	11.05544	40.989982	15.591222	20.60839
Repeat no. 2	11.0222	44	15.00054499	25.7658
Repeat no. 3	13.870011	43.55	13.998882	26.188
Repeat no. 4	11.8749899	45.88442	14.000984	24.0089883
Repeat no. 5	12.08888997	43.98	15.80566	23.301
Repeat no. 6	12.099989911	48.000022	12.000022	27.755

Table S1 (J). Original data for the effects of liraglutide on diabetic cardiac tissue apoptosis showing the percentage (%) of Tunel-positive cells in all experimental groups versus controls.

	Non-diabetic control	Diabetic control	Liraglutide-treated non-diabetic rats	Liraglutide-treated diabetic rats
Repeat no. 1	1.05964000	4.9450079	1.1	2.84999
Repeat no. 2	1.05940000	4.799001	1.21	2.8599519253
Repeat no. 3	1.02500000	3.9004684	1.1001	2.72299931395
Repeat no. 4	1.00001450	4.950034	1	2.7699999
Repeat no. 5	1.00000022	4.950009	1.000067	2.9999999
Repeat no. 6	0.999601097	3.900900976	1	3.0000995999654

Table S1 (K). Original data for the effects of liraglutide on the expression levels of phosporelated-phosphatidylinositol 3-kinase (P-PI3K) proteins.

	Non-diabetic control	Diabetic control	Liraglutide-treated non-diabetic rats	Liraglutide-treated diabetic rats
Repeat no. 1	1.049640000	0.20945779	0.606186431	0.82628538
Repeat no. 2	1.069400000	0.293881038	0.628876416	0.844614653
Repeat no. 3	1.025000000	0.302004684	0.867074738	0.807728952
Repeat no. 4	1.000000145	0.287503425	0.839822067	1.000000209
Repeat no. 5	0.999999822	0.454592778	1.092224111	0.997194908
Repeat no. 6	0.999601097	0.537659331	1.093051149	0.539123917

Table S1 (L). Original data for the effects of liraglutide on the expression of phosphorelated protein kinase B (P-Akt) proteins.

	Non-diabetic control	Diabetic control	Liraglutide-treated non-diabetic rats	Liraglutide-treated diabetic rats
Repeat no. 1	1.011000000	0.3464111	1.422543956	0.802618195
Repeat no. 2	1.210000000	0.37369954	1.670004676	0.887804902
Repeat no. 3	1.000006051	0.307830604	1.363888205	0.826896066
Repeat no. 4	1.011829000	0.347204972	1.451069376	0.843913358
Repeat no. 5	1.000605100	0.247226508	1.085596857	0.862879073

Repeat no. 6	1.000199400	0.265455338	0.948917349	0.659089985

Table S1 (M). Original data for the effects of liraglutide on the expression levels of Integrin-linked kinase (ILK) proteins.

	**Non-diabetic control**	**Diabetic control**	**Liraglutide-treated non-diabetic rats**	**Liraglutide-treated diabetic rats**
Repeat no. 1	0.999968929	0.241855502	0.759337141	1.042872387
Repeat no. 2	1.011948383	0.298206916	0.903088089	1.217802761
Repeat no. 3	0.999937231	0.494280763	0.935759676	0.2181051415
Repeat no. 4	1.000000000	0.333538245	0.3917361904	0.440528673
Repeat no. 5	1.000000000	0.118171806	0.578689427	0.669713656
Repeat no. 6	1.048871078	0.066070264	0.503121797	0.566675985

Table S1 (N). Original data for the effects of liraglutide on the expression levels of phosphorelated phosphatase and Tensin homolog (P-PTEN) proteins.

	**Non-diabetic control**	**Diabetic control**	**Liraglutide-treated non-diabetic rats**	**Liraglutide-treated diabetic rats**
Repeat no. 1	1.11000	4.544248009	1.093053538	0.920418691
Repeat no. 2	1.21000	6.738760382	1.074260416	2.115519253
Repeat no. 3	1.10013	2.575971634	1.145126711	0.949617943
Repeat no. 4	1.00000	6.35362074	1.285996055	4.757678219
Repeat no. 5	1.000067	2.912621626	0.37710956	0.927931395
Repeat no. 6	1.000000	2.237714258	0.487987005	0.790578709
